# Thermogenic crosstalk occurs between adipocytes from different species

**DOI:** 10.1038/s41598-019-50628-9

**Published:** 2019-10-23

**Authors:** Chen Gilor, Kefeng Yang, Aejin Lee, No-Joon Song, Paolo Fadda, Christopher A. Adin, Claire Herbert, Ryan Jennings, Kathleen Ham, James Lee, Ouliana Ziouzenkova

**Affiliations:** 1 Department of Veterinary Clinical Sciences, School of Veterinary Medicine, The Ohio State University, Columbus, Ohio, 43210 USA; 2 Department of Small Animal Clinical Sciences, College of Veterinary Medicine, University of Florida, Gainesville, FL 32610 USA; 3 Department of Medicine and Epidemiology, School of Veterinary Medicine, University of California, Davis. One Shields Ave, Davis, California, 95616 USA; 4 Department of Human Sciences, The Ohio State University, Columbus, Ohio, 43210 USA; 5 Department of Nutrition, School of Medical, Shanghai Jiao Tong University, Shanghai, 200025 China; 6 Genomics Shared Resource, Comprehensive Cancer Center, The Ohio State University, Columbus, Ohio, 43210 USA; 7 Department of Small Animal Clinical Sciences, College of Veterinary Medicine, Michigan State University, East Lansing, MI 48824 USA; 8 Department of Chemical and Biomolecular Engineering, The Ohio State University, Columbus, Ohio, 43210 USA

**Keywords:** Endocrinology, Biotechnology

## Abstract

Visceral obesity increases risks for all-cause mortality worldwide. A small population of thermogenic adipocytes expressing uncoupling protein-1 (*Ucp1*) regulates energy dissipation in white adipose tissue (WAT) depots. Thermogenic adipocytes subsets decrease obesity in mice, but their efficacy has not been tested in obese large animals. Here we enclosed murine subcutaneous adipocytes with and without engineered thermogenic response in biocompatible microcapsules and implanted them into the left and right side of the visceral falciform depot in six obese dogs. After 28 days of treatment, dogs have markedly reduced waist circumference, body weight, and fat mass. *Ucp1* expression in canine WAT was increased at sites implanted with thermogenic *vs*. wild type murine adipocytes. This site-specific thermogenic remodeling of canine tissue by thermogenic murine adipocytes suggests evolutionary conserved paracrine regulation of energy dissipation across species. These findings have translational potential aimed to reduce deleterious WAT depots in humans and pets.

## Introduction

Obesity remains a major worldwide metabolic disorder which poses risks for morbidities, including diabetes, cardiovascular disease, hypertension, cancer, and mortality from these causes^1^. Sedentary lifestyle and dietary choices produce an imbalance in energy intake and expenditure and lead to obesity^2^; overtime, excess of white adipose tissue (WAT) progressively alters endocrine and neurocrine networks, perpetuatuating the obese state^3,4^. These endocrine changes include deregulation of appetite, energy expenditure, glucose metabolism, differentiation and metabolic flexibility in adipocytes in WAT, brown adipose tissue (BAT) and all other tissues^5^. These processes are increasingly dependent on genetic factors that limit treatment options of obesity, maintenance of weight loss, and prevention of co-morbidities in both humans^6^ and pets^7^.

The endocrine contribution of WAT to systemic homeostasis depends on the type of their depots and their mass^8^. WAT depots were initially classified based on the proximity to peripheral organs. Recent tracing studies and gene arrays show evidence that different WAT depots originate from distinct subpopulations of *Myf5*^−^ mesenchymal precursors^9^. Adipocytes from different WAT depots have different heritable genetic and endocrine characteristics^10^. Specifically, visceral abdominal WAT releases adipokines increasing oxidative stress, inflammatory response, and insulin resistance, which increases risk for diabetes, hypertension, cardiovascular disease, carcinogenesis, and overall mortality^1,8^. In addition, visceral WAT has reduced capacity for adaptive lipolysis and thermogenesis compared to the subcutaneous WAT depots^9^. The deficiency in this response is due, in part, to the low numbers of thermogenic adipocytes. These thermogenic adipocytes express uncoupling protein 1 (*Ucp1*) and have high lipolytic and neurocrine activity that release fatty acid from stored triglycerides for energy expenditure^4,9,11^. The reduced presence of thermogenic adipocytes in visceral WAT is likely contributing to the resistance of this depot to weight loss interventions.

In mice, the deleterious properties of visceral WAT could be improved after transplantation of subcutaneous adipocytes into visceral WAT^12^. Even more efficient improvement of metabolism and weight loss was achieved by transplantation of thermogenic adipocytes derived from BAT^13^, thermogenic adipocytes from WAT^11,14^, or engineered thermogenic adipocytes^15^. The limitation of this procedure for treatment of visceral obesity in humans lies in the immune incompatibility of adipocytes with engineered thermogenic responses. To overcome the problem and use genetically modified cells for treatment of human diseases in tissue-specific manner, microencapsulation technology has been under development since 1964 for cells producing insulin for treatment of type 1 diabetes^16^. Recently, our group implemented a semipermeable encapsulation technology for the delivery of thermogenic murine adipocytes into the visceral WAT of obese mice^15^. Biocompatibility between host and transplanted cells is ensured by a nanoporous alginate poly-L-lysine (APL) membrane, which is impermeable to large immunoglobulin molecules. The APL membrane allows for the influx of nutrients and the efflux of endocrine factors and catabolic products^15^. In mice, thermogenic response occurs not only in the encapsulated thermogenic cells, but also in the visceral WAT of the obese host due to the neurocrine induction of thermogenic remodeling^15,17^. Although many genes can affect the thermogenic potential of adipocytes^18^, the silencing of aldehyde dehydrogenase a1 (*Aldh1a1*, alias *Raldh1*) has a unique mechanism of action via production of neuroendocrine factors that mediate global thermogenic remodeling of WAT^17^. This enzyme metabolizes retinaldehydes produced in the vitamin A pathway^19^ with reduced specificity towards other aldehydes^20^ that changes intracellular^15^ and paracrine^21^ thermogenic responses in adipocytes. Although *Aldh1a1* is ubiquitously expressed in all mouse tissues, in adipocytes, deficiency in *Aldh1a1* (*Aldh1a1*^−/−^) increased lipolysis and thermogenesis in visceral WAT and abolished visceral obesity induced by diet or by estrogen deprivation^22^. Notably, encapsulated murine *Aldh1a1*^−/−^ preadipocytes exhibit similar efficacy in the regulation of lipolysis and thermogenesis in adipocytes from different species, as demonstrated in canine adipocytes *in vitro*^21^. To assess the translational potential of subsets of thermogenic adipocytes, it is critical to evaluate their survival, function, biocompatibility, and efficacy in the regulation of energy homeostasis in large animals with a mixed genetic background. In this proof-of-concept study we demonstrate that a small population of thermogenic and subcutaneous murine adipocytes can reduce weight and waist circumference in obese dogs.

## Results

### Microencapsulated murine adipocytes reduced waist circumference and body weight in dogs

**Figure 1:**
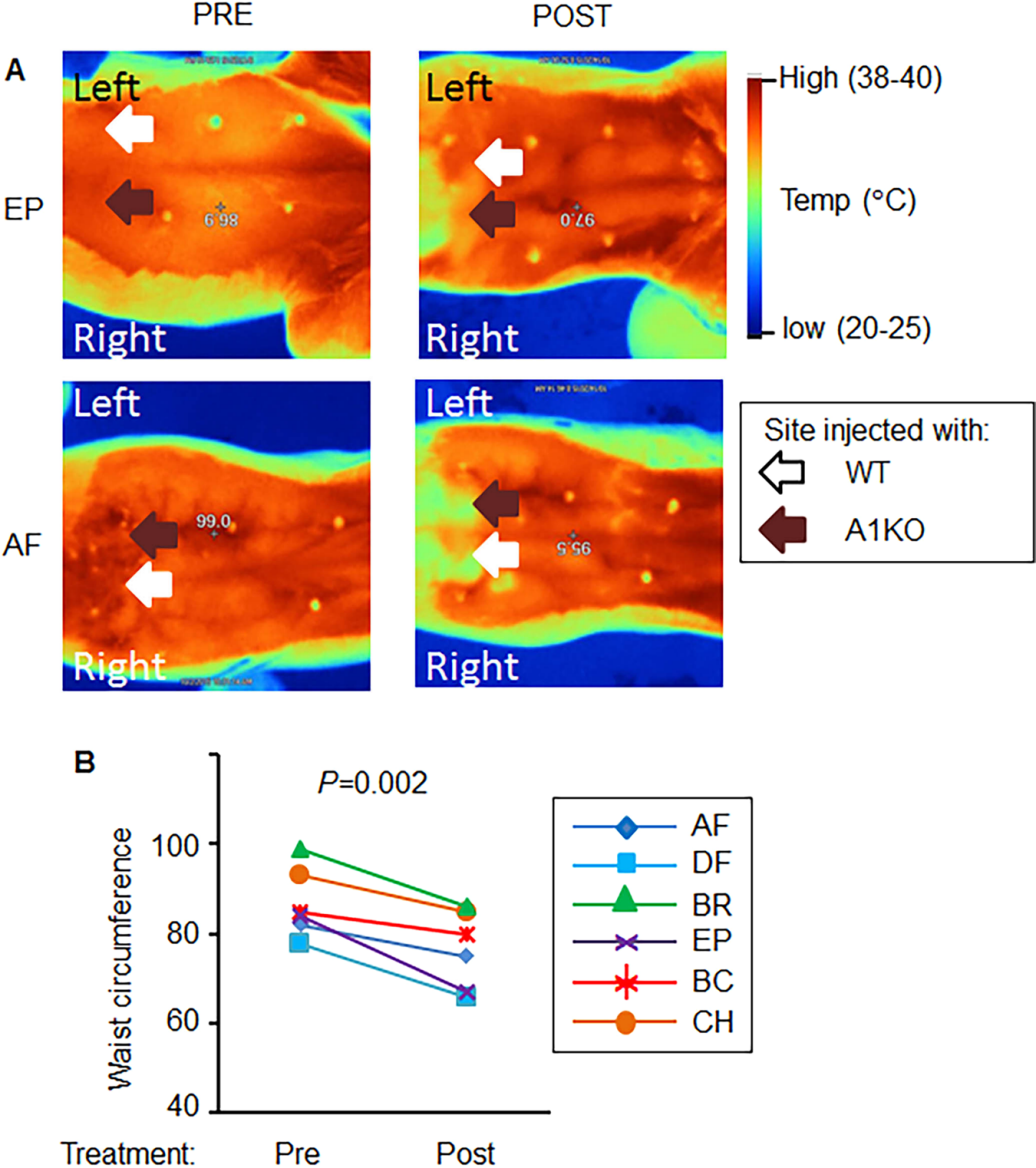
Microencapsulated murine subcutaneous WT and A1KO adipocytes reduced waist circumference. (**A**) The infrared images of dogs (EP, and AF are initials of individual dogs) illustrate the side of injection of encapsulated WT (white arrow) and aldehyde dehydrogenase a1 knock out (A1KO, brown arrow) adipocytes before (Pre) and 28 days after injection (Post). (**B**) Waist circumference (difference between waiste and nipples) was measured in dogs before and 28 days after treatment with encapsulated WT (white arrow) and A1KO as described in (Fig. S1). Paired Student *t*-test.

To prove the concept that minor populations of adipocytes influence metabolic responses across different species, we treated six obese dogs (body fat 34.1 ± 5.9%) with two types of encapsulated mouse adipocytes. Encapsulation in porous APL membrane restricts the exchange of molecules greater that 36kD^15^ between the murine adipocyte xenografts and canine tissues. We compare two types of murine subcutaneous adipocytes (1) Wild type (WT) adipocytes and (2) A1KO adipocytes with thermogenic phenotype that was previously characterized extensively *in vitro* and *in vivo*^15,23^. To eliminate inter-individual variations in responses to these cells among dogs, we performed ultrasound guided injections to insert the encapsulated WT and A1KO cells (10^6^ cells/type/side) on each side of the falciform WAT depot in every dog (n = 6). The infrared images of individual dogs (EP, and AF are initials of two randomly selected dogs) in Fig. 1A show the side of implantation of encapsulated WT (white arrow) and A1KO (brown arrow) adipocytes before and 28 days after implantation. We used these images to calculate relative changes in waist circumference pre and post procedure relative to the permanent position of mammary papillas in dogs (Fig. S1). The implantation of encapsulated mouse adipocytes significantly reduced relative waist circumference post procedure (12 ± 5% range 6–20%) compared to waist circumference before procedure (100%) (Fig. 1B).

**Figure 2:**
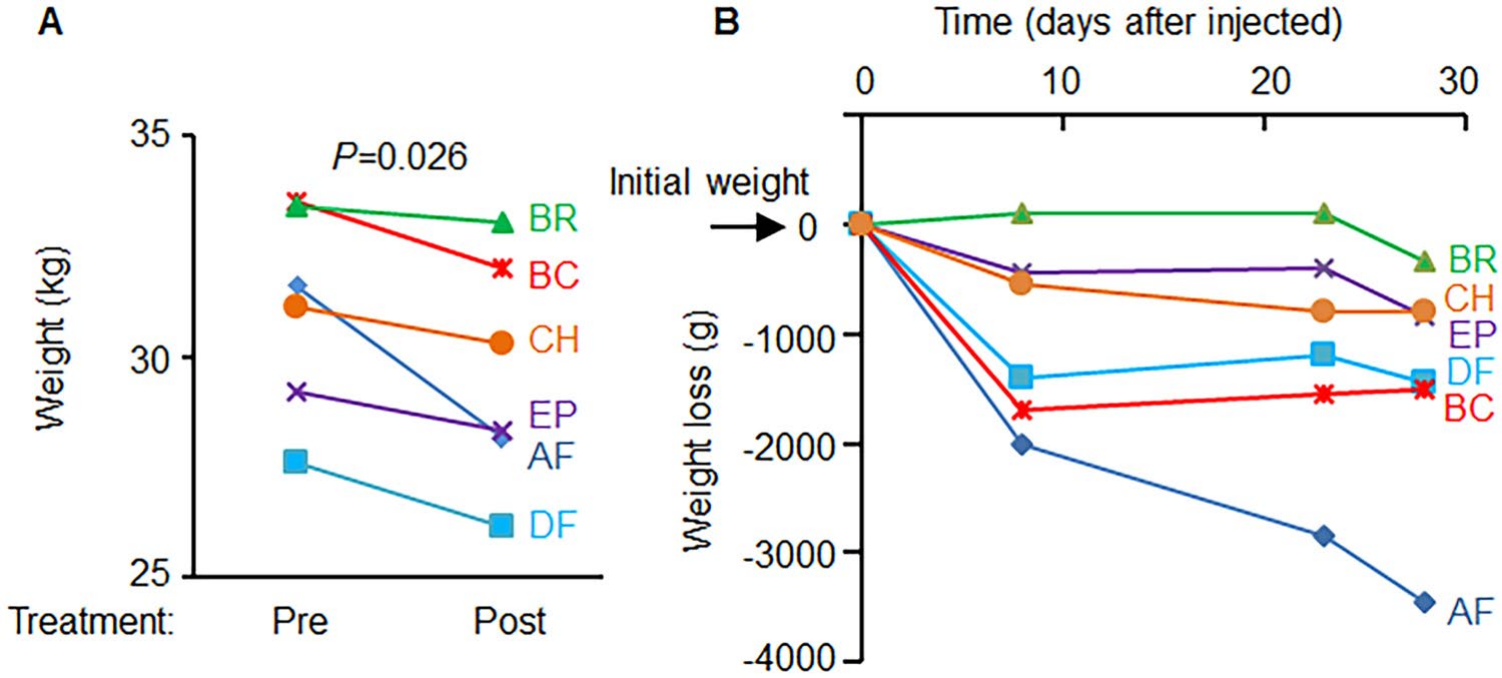
Microencapsulated murine subcutaneous WT and A1KO adipocytes lowered body weight. (**A**) Body weight (kg) was measured before and after treatments. Student paired *t*-test. (**B**) Kinetics of weight loss was shown with body weight measured at day 0 or post 8, 23, and 28 days implantation.

**Figure 3:**
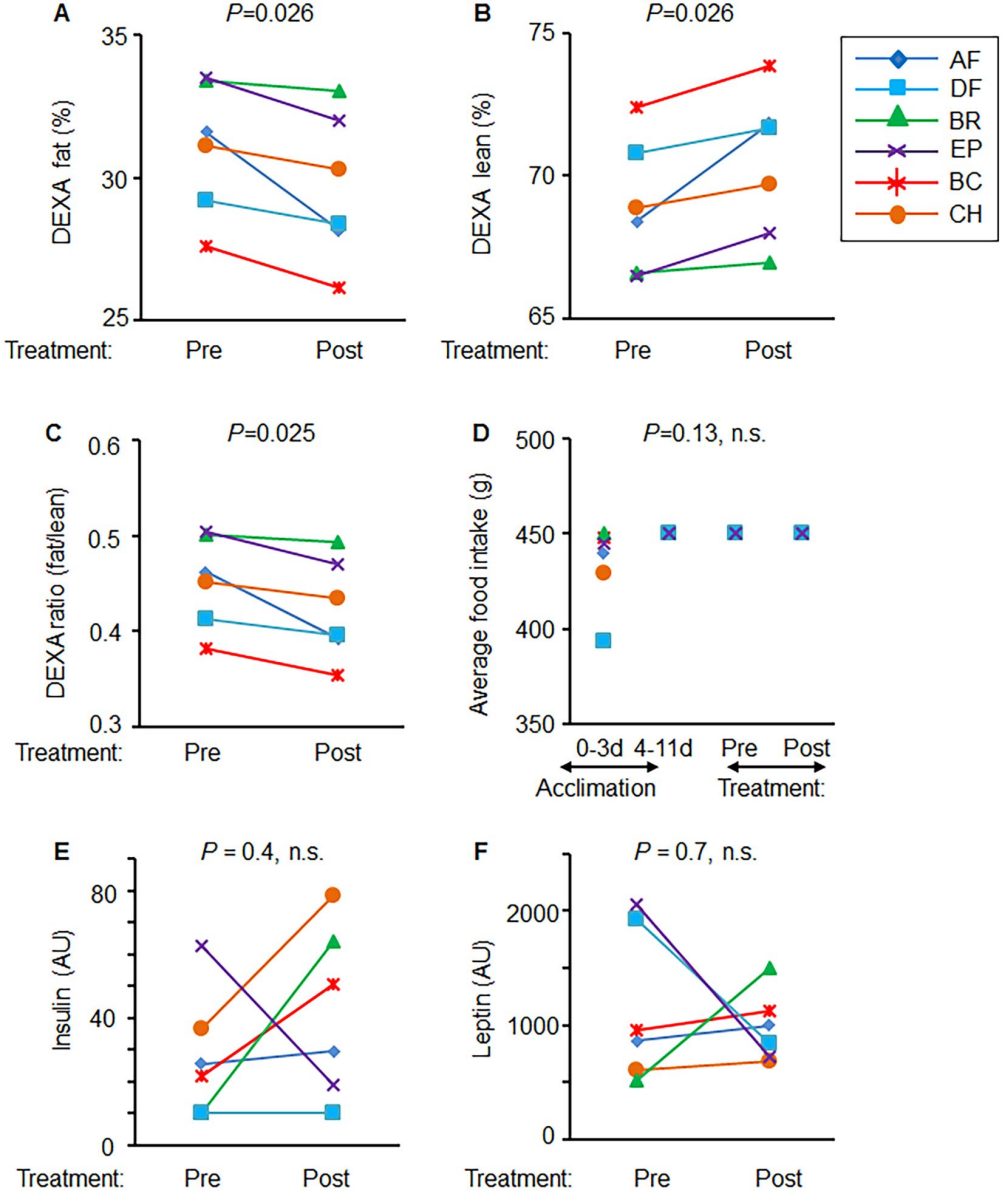
Microencapsulated murine subcutaneous WT and A1KO adipocytes altered fat mass without changes in food intake and levels of insulin and leptin (A–C) Proportion of fat (**A**) and lean mass (**B**) are shown as % of body weight measured by DEXA. (**C**) Fat to lean ratio calculated from these measurements. Student paired *t*-test. DEXA was performed before and 28 days after injection. (**D**) Average food intake of dogs during the acclimation period between day 0 and day 3, and between day 4 and day 11 (total 10 days of acclimation), when dogs’ food consumption became constant (450 g kibble/dog/day). Dogs consumed same identical amount of food 450 g kibble/dog/day during the whole period after injection. Paired Student *t*-test; n.s., not significant. (**E**,**F**) Plasma insulin (**E**) and leptin (**F**) levels were measured before and 28 days after injection. Paired Student *t*-test; n.s., not significant.

The reduction in waist circumference was accompanied by significant weight loss ranging from 1–10.9% (mean ± SD = 4.5 ± 3.5%, *P* = 0.02) (Fig. 2A). The analysis showed weight loss during 28d treatment period (Fig. 2B). On average, dogs have lost 1.4 kg after implantation. The DEXA measurement revealed the significant changes in body fat and lean mass composition (Fig. 3A,B) during 28d treatment period. Dogs have lost a significant amount of fat (*P* = 0.026) while the proportion of lean mass was increased (*P* = 0.026, Fig. 3B). Accordingly, the ratio between fat-to-lean mass was significantly reduced (*P* = 0.025) before and after the treatment (Fig. 3C). These changes in the proportion of fat were not dependent on food consumption, because all animals received and consumed identical amount of provided food throughout the treatment period (Fig. 3D). Before the study, during 3 days of acclimation period (day 0), dogs consumed variable amounts of food. In the subsequent 7 days (total of 10 days of acclimation), each dog consumed the entire amount of food offered daily (450 g/day/dog). During this acclimation 10 days period the body weight in dogs did not change significantly (P = 0.47, ns). Throughout the study dog consumed same amount of kibble (450 g/day/dog).

Insulin and leptin are critical hormones that regulate food intake, fat formation, and glucose metabolism^24^. Plasma levels of insulin (Fig. 3E) and leptin (Fig. 3F) were not altered in dogs in pre and post implantation period. In our study, obese dogs were euglycemic throughout the study (89.8±9 mg/dL vs. 94.3±4 mg/dL before and after study, respectively). This observation is in agreement with other studies finding similar levels of fasting glucose in ‘ideal’ weight dogs 93 (74–128) mg/dL *vs*. 97 (72–135) mg/dL obese dogs^25^.

**Figure 4:**
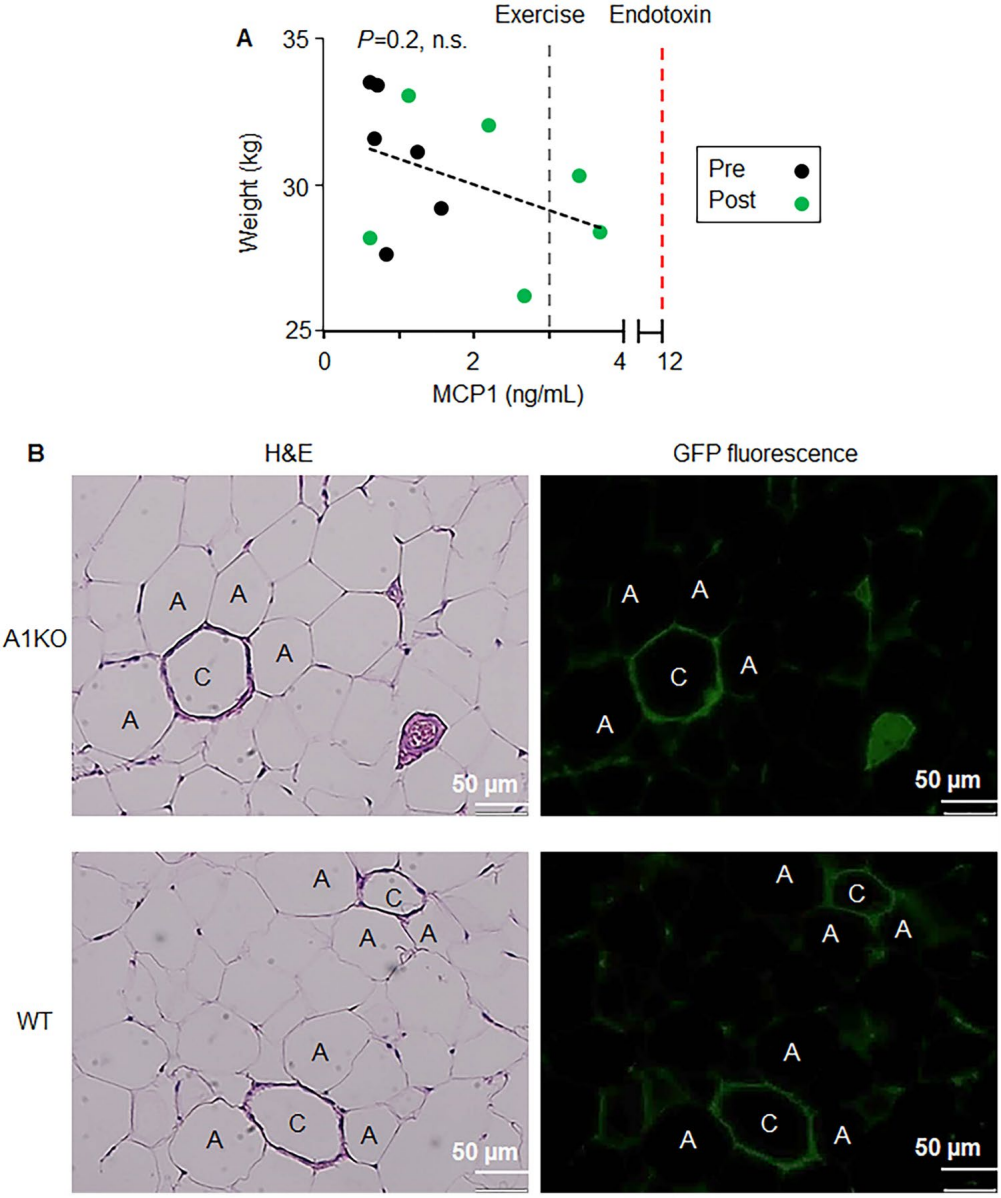
Implantation procedure did not induce pathological inflammatory response in circulation and in dogs adipose tissues. (**A**) A correlation between plasma MCP-1 (Monocyte Chemoattractant Protein-1) and body weight was analyzed. Black dots: before implantation. Green dots: 28 days after implantation. Gray line: criterion for level in exercise. Red line: criterion for endotoxin level. Pearson correlation coefficient; n.s. not significant. (**B**) Histological examination was performed in dogs adipose tissues stained with H&E. A1KO: aldehyde dehydrogenase a1 (*Aldh1a1*) knock out adipocytes. WT: wild type murine adipocytes. A: Adipocyte, C: microcapsule.

We also measured the level of inflammatory cytokines in a multiplex format before and after implantation to assess the cumulative influence of the implantation procedure, biocompatibility of xenogeneic transplantation, and/or or the effect of adipose tissue loss. The levels of MCP1 in plasma were increased post procedure (Fig. 4A, black vs. green circles); however, this increase did not correlate with dogs’ weight. Dogs exhibited a wide range of changes in MCP1 before and after treatment. In Fig. 4A, we indicated the range of published values for the MCP1 levels in canine plasma in response to physiologic stimulation by endurance exercise^26^ as well as pathologic levels after exposure to endotoxin^27^ using the same assay. Similar relationships were also demonstrated for the other inflammatory cytokines, such as IL-6, GM-CSF, and TNFα (Fig. S2). All levels of tested inflammatory cytokines were below published levels induced by endotoxins^27^. None of the tested inflammatory markers correlated with body weight.

The histological examination of adipose tissue samples revealed that in the intact WAT, microcapsules (Fig. 4B, green-fluorescence indicates encapsulated cells) were surrounded by adipocytes, indicating the absence of inflammatory response and fibrosis. However, both encapsulated cells and inflammatory cells were present in other specimens of WAT suggesting that ultrasound-guided injection and damage of some capsules could contribute to the mild inflammatory response seen 28d after the treatment.

### Encapsulated thermogenic adipocytes increase *Ucp1* expression levels in canine WAT

**Figure 5:**
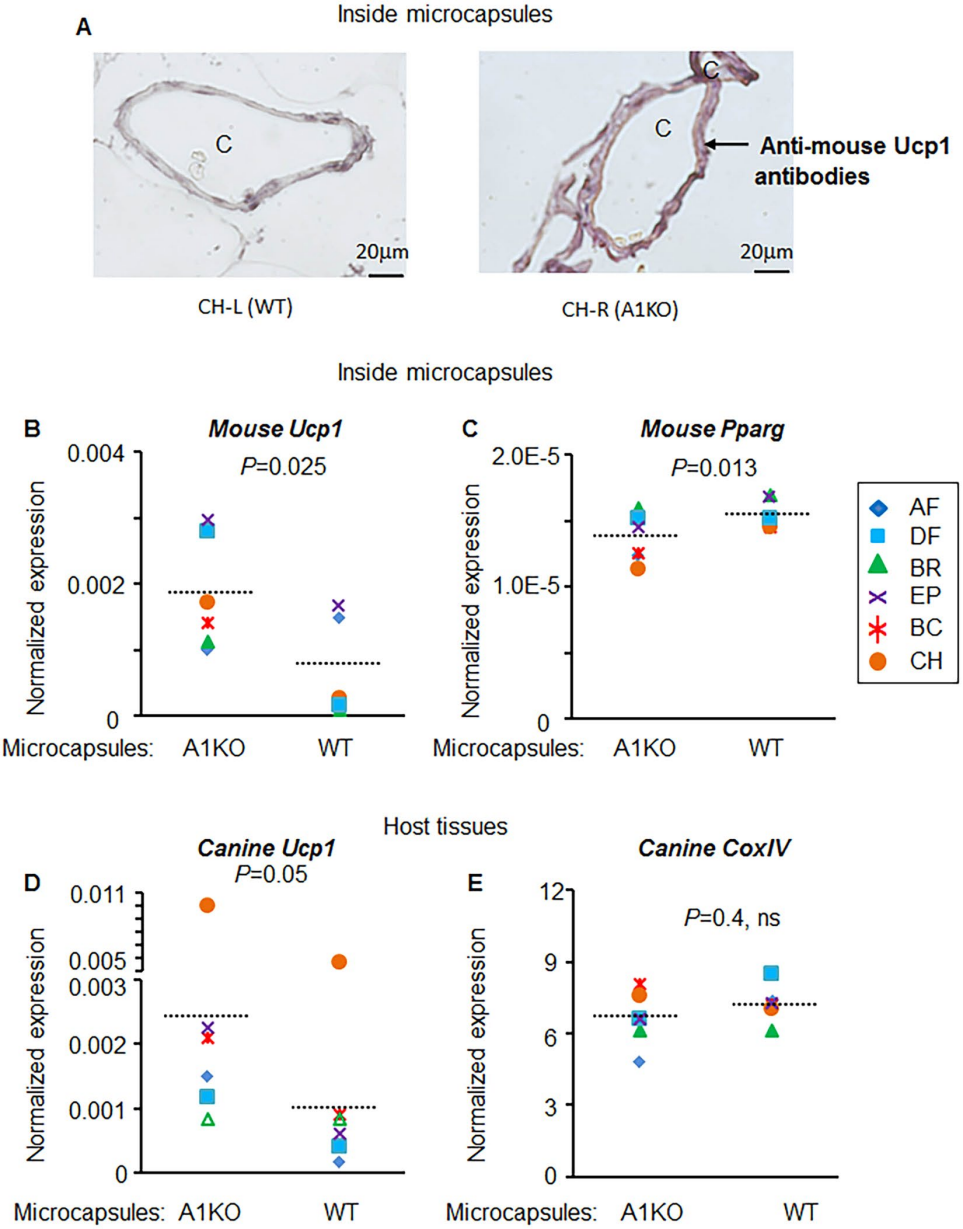
Transplanted murine A1KO adipocytes induced expressions of mouse and canine *Ucp1* and mouse *Pparg*. (**A**) UCP1 expression (brown staining) in a representative example of a dog (CH) paraffin embedded adipose tissues. A1KO: aldehyde dehydrogenase a1 (*Aldh1a1*) knock out adipocytes. WT: wild type murine adipocytes. C: microcapsule. (**B–E**) Normalized expressions of mouse (inside microcapsules) or canine (host tissues) genes were compared between A1KO (aldehyde dehydrogenase a1 (*Aldh1a1*) knock out) cell-implanted or WT (control wild type) cell-implanted region (left or right region of the same dog). Student paired *t*-test. *Ucp1*, uncoupling protein 1 (**B**,**D**); *Pparg*, Peroxisome proliferator-activated receptor gamma (**C**); *CoxIV*, Mitochondrial cytochrome c oxidase subunit IV (**E**).

Increased expression of thermogenic gene *Ucp1* is a well-established factor leading to reduction of obesity^9^. We investigated the effects of treatment with encapsulated murine cells on the adipogenic and thermogenic gene expression of canine WAT. The gene expression was measured in WAT biopsies dissected from the left and right side of the same WAT depot treated with encapsulated WT or A1KO murine adipocytes. Gene expression was selected as a preferred quantitative method distinguishing between the murine and canine *Ucp1* genes. Antibodies do not exhibit the required species specificity, although mouse UCP1 protein was expressed within capsules containing A1KO *vs*. WT cells (Fig. 5A). We found that the expression of mouse *Ucp1* was significantly greater in WAT transplanted with A1KO compared to WT adipocyte microcapsules (935% vs. 100%, Fig. 5B). In contrast, the expression of peroxisome proliferator-activated receptor (*Pparg*), a master regulator of adipogenesis in WAT, was significantly reduced in WAT transplanted with A1KO compared to WT adipocyte microcapsules (Fig. 5C). These data are in agreement with the previously reported^15,28^ higher levels of *Ucp1* accompanied by lower levels of *Pparg* in A1KO vs. WT adipocytes. The expression of murine genes also demonstrates the survival of functional encapsulated mouse cells in canine tissues 28 days after implantation.

The treatment also increased expression of canine *Ucp1* in WAT treated with encapsulated A1KO (343%) vs. WT adipocytes (100%) (Fig. 5D). The expression levels of other mitochondrial genes, such as mitochondrial gene cytochrome c oxidase subunit 4I1 (*CoxIV*) remained at similar levels in dogs treated with encapsulated A1KO and WT adipocytes (Fig. 5E). These data indicate that encapsulated A1KO cells influence local thermogenic remodeling of the canine WAT.

**Figure 6:**
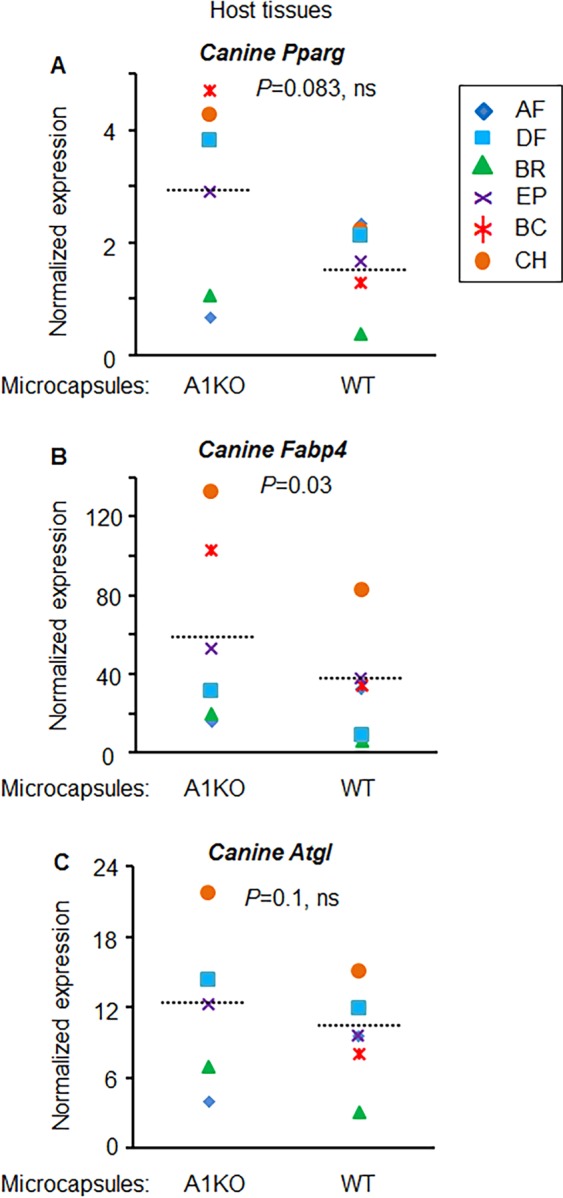
Implanted murine A1KO adipocytes influenced expressions of host *Pparg* and its target genes. (**A**–**C**) Normalized expressions of canine (host tissues) genes were compared between A1KO (aldehyde dehydrogenase a1 (*Aldh1a1*) knock out) cell-implanted or WT (control wild type) cell-implanted region (left or right region of the same dog). Paired Student *t*-test. *Pparg*, Peroxisome proliferator-activated receptor gamma (**A**); *Fabp4*, fatty acid binding protein 4 (**B**); *Atgl*, Adipose triglyceride lipase (**C**).

**Figure 7:**
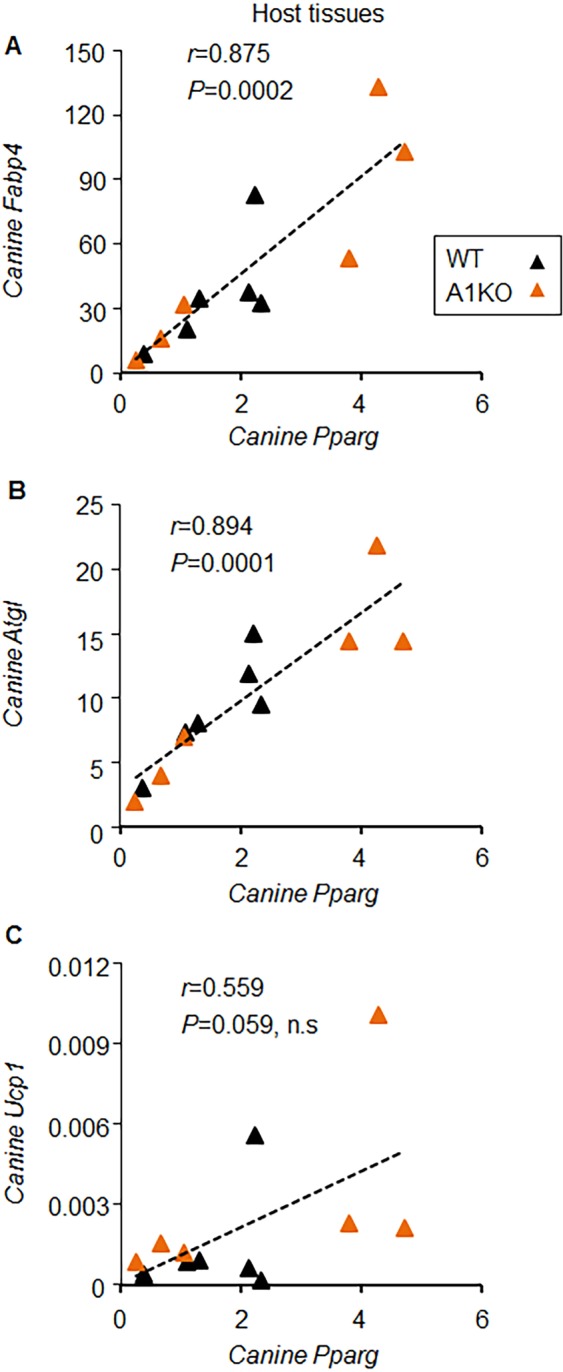
Implanted murine A1KO adipocytes influenced host *Pparg* expression correlating with expressions of its target genes. (**A**–**C**) Correlation was tested with normalized canine *Pparg* expression and *Fabp4* (**A**), *Atgl* (**B**), and *Ucp1* (**C**), respectively. Black triangle: WT (wild type). Orange triangle: A1KO (aldehyde dehydrogenase a1 (*Aldh1a1*) knock out). Pearson correlation coefficient. *Pparg*, Peroxisome proliferator-activated receptor gamma; *Fabp4*, fatty acid binding protein 4; *Atgl*, Adipose triglyceride lipase; *Ucp1*, uncoupling protein 1.

We also analyzed the expression of canine *Pparg* and its two target genes: fatty acid binding protein 4 (*Fabp4 or aP2*)^29^ and adipose triglyceride lipase (*Atgl* or *Pnpla2*)^30^. The changes in *Pparg* levels did not reach statistical significance (Fig. 6A). *Fabp4* expression was significantly increased in canine WAT treated with encapsulated A1KO compared to WT adipocytes (Fig. 6B), but *Atgl* expression was similar in these samples (Fig. 6C). Both *Fabp4* and *Atgl* levels significantly correlated with the levels of *Pparg* in the same tissue (Fig. 7A,B), in agreement with regulation of their transcription by *Pparg*. In contrast the expression of *Ucp1* was not significantly correlated with *Pparg* (Fig. 7C). Thus, genetic modification of encapsulated cells (A1KO, but not WT) can induce specific thermogenic remodeling processes in the treated locus of WAT depot.

## Discussion

Here we demonstrate that implantation of a small subset of encapsulated A1KO adipocytes into the visceral fat of obese dogs induced local thermogenic remodeling of visceral fat. This response was side-specific, because encapsulated WT subcutaneous adipocytes implanted on the other side of the same fat depot lacked this effect. This asymmetric thermogenic remodeling in response to implanted A1KO vs. WT adipocytes shows the feasibility of using engineered cells for modification of local gene expression and functional responses in specific tissues and their compartments. Among fat depots, visceral fat expansion poses the greatest risk for morbidity and all-cause mortality^1^. Attainment of therapeutic effects in specific tissues will advance treatment of metabolic degenerative diseases in the future.

In our study, genetic A1KO makeup played an important role in both activating intracellular thermogenic programs in A1KO adipocytes as well as inducing gene expression in host canine adipose tissue. Similar effects were previously reported *in vitro*, using co-cultures of murine A1KO and canine adipocytes^21^. Although many genes have been proposed to induce thermogenic gene expression programs^9,31,32^, the phenotype of A1KO adipocytes is uniquely suitable for application in biocompatible microcapsules. A1KO cells maintain constitutive thermogenic characteristics in cell culture as well as after their implantation into adipose tissue of obese hosts^15^. Natural beige and brown adipocytes required sympathetic activation^9^ or other factors, *e*.*g*. nutrient^31^ or cytokine stimulants^33^, to activate thermogenesis. These types of thermogenic responses are transient and can be further suppressed in obesogenic environments. A1KO adipocytes utilize evolutionarily conserved lipid mediators to regulate thermogenic programs in genetically diverse organisms, including dogs from different breeds^21^ and WT mice^15^. In adipocytes, *Aldh1a1* (A1) controls production of retinoic acid from retinaldehyde to regulate expression of *Pparg*^28^, whereas in A1KO adipocytes, retinadehyde blocks *Pparg* activation and induces thermogenic programs^22,28^. A1KO adipocytes secrete signaling molecules through the nanoporous capsules for activation of thermogenic programs in canine adipocytes^21^. This paracrine mechanism is supported by proximal expression of canine *Ucp1* in adipose tissues with implanted A1KO but not with WT adipocytes. The amplification of the thermogenic responses in the host WAT is likely responsible for efficacy of this relatively small number of encapsulated A1KO adipocytes.

Treatment with WT and A1KO cells changed systemic metabolic responses, manifesting as marked weight loss, decrease in fat mass, and a reduction in waist circumference. These changes resulted in a decrease in fat to lean mass ratio. The limitation of our pilot study was in the short duration of the treatment, which was designed to assess early changes in gene expression. Particularly, dogs with the highest content of fat (*i*.*e*. BR) responded to the treatment with weight loss only after 20 days, suggesting that a longer treatment period could improve therapeutic outcomes. The design of our studies with implantation of both WT and A1KO cells did not allow us to draw any causative conclusions of the contribution of subcutaneous and/or thermogenic adipocytes to the loss of weight and fat mass in the host animals. However, this and other systemic effects are in agreement with multiple other studies reporting weight loss after transplantation of subcutaneous^12^ and thermogenic adipocytes into WAT^11,13–15^. In future studies, systematic bioenergetic characterization of host tissues needs to be performed to identify key factors responsible for systemic changes in the host organism.

Inflammation, both chronic and acute, is a critical confounding process influencing thermogenesis and obesity^33^. In our study, obesity^8^ and xenobiotic response^34^ are potential sources of chronic inflammation, whereas ultrasound-guided implantation could increase acute inflammatory response. While injection of cells into fat depot might cause local cell damage, this procedure is minimally invasive and is unlikely to cause acute systemic inflammation. We found that the levels of inflammatory cytokines after treatment were moderately increased at 4 weeks post treatment. These cytokine levels were consistent with the reported levels induced by endurance exercise^26^ and markedly lower than reported levels of these cytokines induced by endotoxins^2^. In our study, this inflammatory process did not critically contribute to thermogenic remodeling, because *Ucp1* expression was increased in the WAT areas treated with encapsulated A1KO adipocytes but not with WT adipocytes. The systemic inflammatory response is expected to increase thermogenesis in whole WAT depot^33^. These changes in the levels of inflammatory cytokines did not correlate with weight changes, suggesting that post-injection inflammation was not the major factor influencing systemic metabolic responses in this study. Regardless, inflammation is an adverse effect of the technique used in the current study and the implantation procedure leading to partial rupture of capsules should be refined for veterinary and clinical applications in the future, with a focus on minimizing the rupture of the capsules during implantation. Potential refinements of both encapsulation hydrogels and coatings and implantation procedure have been developed to improve stability of microcapsules, limit inflammation, and improve functional survival of encapsulated cells for weeks in mice and primates, and for years in humans^35,36^. For the future translation of this technology the examination of long-term safety and stability of microcapsules as well as their efficacy in the WAT milieu is the primary direction of research. One major advance made in our study was validation of the efficacy of xenogenic A1KO genotype for the efficient induction of thermogenesis in visceral WAT in large animal models.

Previous studies demonstrate the efficacy of thermogenic adipocytes for improvement of metabolic response in genetically uniform small animal models^11,13–15^. The major translational challenge was to extrapolate functional and therapeutic relevance of this small population of cells to patients. Metabolic diseases in humans and other large mammalians, including dogs is multifactorial and develop as a result of complex interactions of genetic, endocrine, and environmental factors^5,37^. Here we demonstrate for the first time that a small subset of differentiated cells, such as adipocytes, can lead to thermogenic modification of adipose tissue in obese dogs.

This study demonstrates that site-specific thermogenic remodeling of visceral fat depot can be achieved by implantation of a small subset of encapsulated murine thermogenic adipocytes into abdominal WAT in a large animal model of obesity, such as obese dogs. The genetic deficiency in *Aldh1a1* gene of adipocytes is sufficient to a maintain thermogenic phenotype within the murine adipocyte and stimulate paracrine induction of *Ucp1* expression in canine adipocytes *in vivo*, demonstrating that pathways for regulation of energy expenditure are conserved among species. In response to treatment with encapsulated subcutaneous WT and A1KO adipocytes, obese dogs had a significant decrease in fat mass, whole body weight, and waist circumference. Although the causal relationship between the thermogenic effects of A1KO adipocytes and weight loss need to be elucidated in future studies, biocompatible encapsulated adipocytes might provide a new strategy for cost-effective and long-term solution for alleviation of metabolic conditions in large mammals and, possible translational development of this technology for obese patients.

## Materials and Methods

All animal use was approved by the Ohio State University Institutional Animal Care and Use Committee OSU-IACUC (Protocol 2012A00000147). All methods were performed in accordance with the relevant guidelines and regulations.

### Preparation of encapsulated cells for injections

Wild type (WT) C57BL/6 J and alcohol dehydrogenase 1a1 deficient (*Aldh1a1*^−/−^) female mice were used to dissect subcutaneous WAT depots and isolate stromal vascular fraction. From these cells we derived WT and *Aldh1a1*^−/−^ (A1KO) fibroblast cell lines labeled with green fluorescence protein using canonic immortalization procedure and stable transfection as previously described^15,23^. Prior to transplantation, cells were encapsulated into alginate-poly-L-lysine (APL) using protocol^23^ for the phase microencapsulation technique. The cell suspension (2 × 10^6^ cells/ml/batch) was suspended in 2% sodium alginate solution (Sigma, St. Louis, MO, MW 12,000–80,000 g/mol, 100–300 cps Brookfield viscosity). Then, this suspension was extruded into a 100 mM CaCl_2_ solution, using Encapsulation Unit (Nisco Engineering AG, Switzerland) at 5.4 kV to form calcium alginate gel beads. Nozzle size, flow rate, and vibration frequency were identical for all encapsulation experiments. The gel beads trapped with cells were solidified in 100 mM CaCl_2_ for 20 min, and then incubated with 0.05% (w/v) poly-L-lysine (MW 20,700, Sigma, St. Louis, MO) to form a transparent alginate-poly-L-lysine membrane around the surface. The membrane-enclosed gel beads were further suspended in 50 mM sodium citrate to liquefy the alginate core. Floating cultures of encapsulated fibroblasts were cultured in standard medium DMEM containing 10% calf serum and 1% penicillin/streptomycin and were maintained in culture under standard conditions for up to 30 days.

### Dogs and treatment

Six purpose-bred, healthy intact female mixed breed hounds, aged 1.7 ± 0.3 years (median 1.75, range 1.25–2.0) were used in this study. The obesity in these dogs is seemingly the result of long standing overfeeding paired with minimal exercise. Dogs were housed in individual runs, with visual interaction with other dogs, in an AAALAC-accredited facility for the duration of the study. All dogs were housed in a single room. Dogs were fed a standard maintenance dog kibble (3.5 kcal/g Laboratory Canine Diet 5006; LabDiet; St. Louis, MS, USA). A fixed amount of kibble (~450 g, 1575 kcal) was offered once daily at 7:00 am and the uneaten portion was weighed and recorded. Dogs were acclimated to the lab for 11d prior to the beginning of the experiment. In the first 3d of acclimation, food consumption vartied. From the 4^th^ day to 11 days of acclimation each dog consumed its daily meal in its entirety (total 10 days of acclimation). After food consumption was consistent, study began and throughout the study, each dog consumed its daily 450 g of kibble entirely. Throughout the study, body weight was recorded weekly. No oral supplements, medications, or treats were administered from one week prior to study initiation through study completion; no changes in caloric allocation were made, regardless of body condition score at the beginning of the study.

*Procedure*: On day 1, dogs were sedated (dexdomitor 0.009 mcg/kg intramuscular (IM), butorphanol 0.2 mg/kg IM), blood samples were collected and DEXA scans were performed using the GE Lunar Prodigy (Fairfield, CT). Percent body fat was measured using enCORE software (Fairfield, CT). Then, the falciform fat of each dog was injected with encapsulated A1KO on one side and with encapsulated WT fibroblasts on the other side. Sides were chosen randomly and blinded for the personnel. Injection of the encapsulated cells was performed using ultrasound guidance, with the needle entering the skin 4 cm cranial to the umbilicus and 2 cm lateral to midline. The needle was inserted to a depth of 2 cm into the falciform fat and maintained in that position while the injection was completed. The sedation was then reversed and the dogs were returned to their kennels and resumed their daily routine. Twenty eight days later, the DEXA scan and blood tests were repeated under sedation. The next day the dogs were spayed and the falciform fat was removed in its entirety, weighed, and divided to left and right side.

### Blood collection, processing and analysis

Blood glucose concentrations were measured with a hand-held point-of-care glucose monitor that was previously validated for use in dogs (AlphaTRAK 2; Abbott Animal Health, Abbott Park, IL, USA). Blood samples for cytokine and hormone analysis were collected into chilled EDTA tubes and plasma was separated within 2 h.

Plasma was stored in −20° C and was analyzed in one batch at the end of the study. The analysis was performed in blind fashion. Plasma levels of canine IL6, MCP1, GMCSF, TNFα, insulin, and leptin were measured with MILLIPLEX MAP Canine Cytokine/Chemokine and Canine Endocrine Magnetic Bead Panels, Millipore/Merck, Darmstadt, Germany, Cat No CCYTOMAG-98K). All samples were tested in duplicates.

Paraffin embedded sections of right and left side of injected falciform fat were used for hematoxylin and eosin staining (H&E), as well as immunohistochemistry analysis using rabbit polyclonal antibodies against mouse UCP1 (Abcam, ab10983, react with canine and mouse UCP1) was performed as previously descriebed^15^. Antibody was used at 1:1000 dilution.

### mRNA analysis

Total mRNA was purified from canine adipose tissue containing encapsulated murine WT or A1KO fibroblasts according to the manufacturer’s instructions (Qiagen, Germantown, MD). RNA integrity was analyzed using the Agilent 2100 Bioanalyzer (Agilent Technologies, Santa Clara, CA). mRNA was quantified using 12 K Flex QuantStudio Real-Time PCR System and TaqMan fluorogenic detection system (Applied Biosystems/ThermoFisher). To examine expression of canine genes, we used validated *Ucp1* (Cf02622090_m1), LOC479623 (Cf02644082_m1), *Pparg* (Cf02625640_m1), *Atgl* (PNPLA2 Cf0260386_g1), *Fabp4* (AIVI5UH) primers; which were also purchased from ThermoFisher. Simultaneously, in the same samples we also analyzed mouse *Ucp1* (Mm01244861_m1). Comparative real-time PCR was performed in triplicate, including no-template controls. Expression was calculated using the comparative Ct method normalized to the TATA box binding protein (TBP: canine Cf02637234_m1 and murine Mm00446973_m1).

### Statistical analysis

All data are shown as mean ± SD. Number of samples is indicated in Figure legends. Group comparisons were assessed using Paired Student *t*-test. The statistical relationship between two continuous variables was measured by Pearson test. *P* < 0.05 was considered to be statistically significant.

## Supplementary information

Supplementary Info

## Data Availability

All the data for this study are present in the main text or in the Supplementary Materials.
