# Supplementary material for: Thermogenic crosstalk occurs between adipocytes from different species
Source: Sci Rep. 2019 Oct 23;9:15177. doi: 10.1038/s41598-019-50628-9 (PMC6811532; doi:10.1038/s41598-019-50628-9)

Thermogenic crosstalk occurs between adipocytes from different species.

Chen Gilor, Kefeng Yang, Aejin Lee, No-Joon Song, Paolo Fadda, Christopher A. Adin, Claire Herbert, Ryan Jennings, Kathleen Ham, James Lee, Ouliana Ziouzenkova.

Supplementary Information

fig. S1. Waist circumference measurements in dogs treated with microencapsulated murine adipocytes.

(**A**) The total waist circumference that was localized in the proximity to visible mammary papillas in dogs, was indicated using infrared images of dogs (EP is initial of an individual dog) before and after implantation procedure. The dog were not sedated for imaging, therefore, we used the most visible pair of papillas in each dog.

(**B**) Changes in waist circumference were presented after subtraction of distance between mammary papillas from the total waist circumference in each dog.

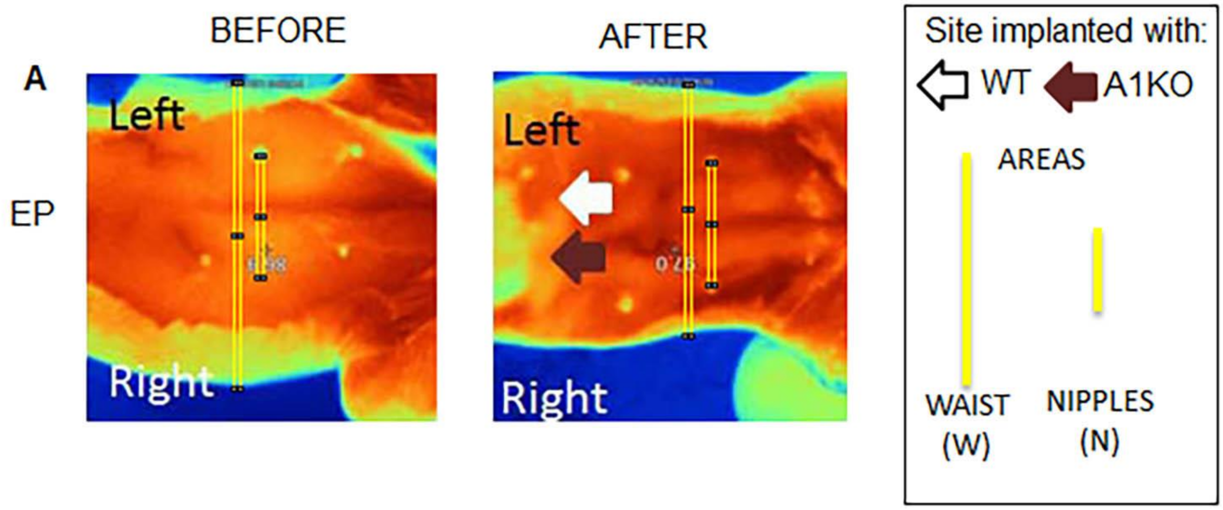

B

	Total waist area (W)	Distance between nipples (N)	**Differences (W-N)**
EP (before)	146	58	**88**
EP (after)	119	58	**61**

fig. S2. Lack of association between weight loss and the levels of inflammatory cytokines in circulation in dogs treated with murine adipocytes (**A-C**) A correlation between body weight and plasma interleukin-6 (IL-6, **A**), granulocyte-macrophage colony-stimulating factor (GM-CSF, **B**), and tumor necrosis factor-α (TNFα, **C**) levels before (black circles) and 28d after implantation (green circles). Dotted lines: published levels of cytokines induced by exercise (grey) or endotoxin (red), for GM-CSF these data were not available), n/a. Pearson correlation coefficient; n.s. not significant.

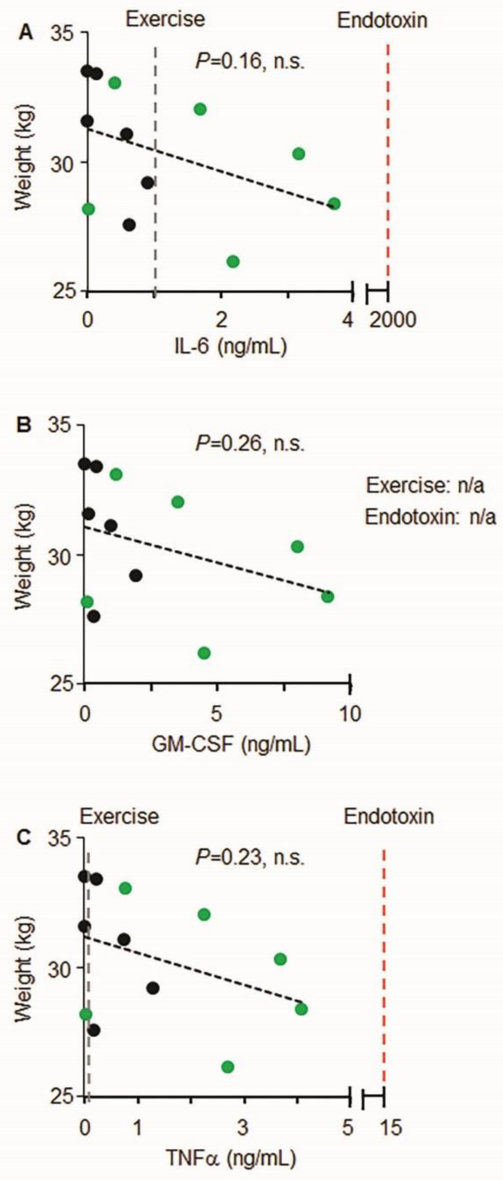

Supplement: Supplementary file 1 — Supplementary Info [file 41598_2019_50628_MOESM1_ESM.pdf]
